# A novel ferroptosis-related 12-gene signature predicts clinical prognosis and reveals immune relevancy in clear cell renal cell carcinoma

**DOI:** 10.1186/s12885-021-08559-0

**Published:** 2021-07-19

**Authors:** Yingkai Hong, Mingen Lin, Dehua Ou, Zhuangkai Huang, Peilin Shen

**Affiliations:** 1 Department of Urology, The First Affiliated Hospital of Shantou University Medical College, Shantou, People’s Republic of China 515041; 2 Shantou University Medical College, Shantou, People’s Republic of China

## Abstract

**Background:**

Clear cell renal cell carcinoma (ccRCC) is still highly aggressive and lethal even with various therapeutic approaches. As the kidney is an iron metabolism-related organ, exploring and assessing the clinical value of ferroptosis, an iron-dependent regulated cell death, is practical and important.

**Methods:**

Prognostic ferroptosis-related differentially expressed genes (DEGs) were identified from the KIRC cohort in the cancer genome atlas (TCGA) database, from which a prognostic signature was established using Lasso-penalized Cox regression analysis. Each patient in the KIRC cohort and the E-MTAB-1980 cohort (from the ArrayExpress database) was assigned a calculated signature-correlated risk score and categorized to be either in the high- or low-risk group divided by the median risk score in the KIRC cohort. Then, the independent prognostic value of the signature was further assessed by Kaplan-Meier (K-M) survival, time-dependent receiver operating characteristic (ROC) and Cox regression analyses based on overall survival (OS) in both cohorts. Finally, risk-related DEGs were identified in both cohorts and subjected to enrichment analyses for Gene Ontology (GO), Kyoto Encyclopedia of Genes and Genomes (KEGG) and immune infiltration.

**Results:**

Among 60 ferroptosis-related genes, 32 prognostic DEGs were identified, from which we constructed a prognostic 12-gene signature with CARS1, HMGCR, CHAC1, GOT1, CD44, STEAP3, AKR1C1, CBS, DPP4, FANCD2, SLC1A5 and NCOA4. Patients in both cohorts were divided into high- and low-risk groups, which were visually distributed in two sets and had positive-risk-related mortality. The K-M survival and the ROC curves validated that the signature has prognostic value with *P* < 0.05 and area under the curve > 0.7 in both cohorts, respectively. Multivariate Cox regression further confirmed the risk score as an independent prognostic predictor for OS. Commonly enriched terms in GO and KEGG not only showed a high iron correlation but also, interestingly, immune relevance of 3 immune cells (macrophages, mast cells and regulatory T cells) and 1 immune-related function (antigen processing cell co-stimulation).

**Conclusion:**

We established a novel 12 ferroptosis-related-gene signature that was proven to be an independent prognostic predictor for OS and inferred to be related to tumour immunity in ccRCC; however, the underlying mechanism is still poorly characterized and needs further exploration.

**Supplementary Information:**

The online version contains supplementary material available at 10.1186/s12885-021-08559-0.

## Background

As the latest research suggests, renal cell carcinoma (RCC) is the second most commonly diagnosed urological cancer after bladder cancer, in which approximately 80% are clear cell RCCs (ccRCCs) [1, 2]. Even with various therapeutic approaches, such as surgery, chemotherapy, radiotherapy, targeted therapy and the newly proposed immunotherapy, ccRCC is still one of the most difficult clinical problems in urology. Delays in diagnosis and a high metastatic rate are the main causes. The incidence of advanced ccRCC is approximately 33% at patients’ first hospital visits, and 40% develop distant metastases and suffer from poor survival outcomes (the 5-year survival rate is less than 11.2%) [3]. For localized RCC, radical nephrectomy is still the major treatment modality. Concerning metastatic tumours, conventional therapeutic methods such as multitarget tyrosine kinase inhibitors (TKIs) and mammalian target of rapamycin (mTOR) inhibitors are extensively adopted; however, the therapeutic benefits are modest [4].

Iron is an essential element in the basic biological processes of the human body, and metabolic disorders are involved in the occurrence and progression of many tumours [5, 6]. Recently, iron-dependent regulated cell death (RCD), namely, ferroptosis, has drawn increasing attention in the cellular-molecular field of tumours. Less than a decade ago, ferroptosis was introduced as a nonapoptotic RCD distinguished from necroptosis [7], pyroptosis [8], and alkaliptosis [9, 10]. In 2012, Dixon et al. [11] first demonstrated that in contrast to apoptotic inhibitors, the growth inhibitory effect of erastin on RAS-mutant cancer cells can be completely antagonized by iron chelators and lipophilic antioxidants relying on a new form of RCD named ferroptosis. Morphologically, unlike typical apoptotic features such as membrane blebbing and shrinkage, classical necrosis-like features such as cell swelling and plasma membrane rupture can be observed during ferroptosis [12]. Biochemically, ferroptosis is driven by reactive oxygen species (ROS), which are highly associated with iron accumulation and lipid peroxidation [13]. Due to their high metabolic characteristics, most tumours are in a state of high oxidative stress and are required to increase their ROS scavenging ability to prevent oxidative damage, which may make them sensitive to ferroptosis [14]. Many cancers have been proven to be ferroptosis-related, such as hepatocellular carcinoma [15], gastric cancer [16, 17], ovarian cancer [18, 19], and breast cancer [20, 21]. Therefore, inducing ferroptosis to promote cell death or inhibit cell growth for cancer could be a new therapeutic strategy [22].

The kidney is an iron metabolism-related organ with biofunctions, such as balancing iron homeostasis by filtering and reabsorbing iron and promoting haemoglobin synthesis by forming erythropoietin [23]. Several studies have demonstrated that ccRCC is highly associated with iron metabolism [24, 25]. However, the role of ferroptosis in ccRCC remains poorly understood. Thus, exploring the potential correlation between ccRCC and ferroptosis is practical and important.

To explore and assess the clinical value of ferroptosis in ccRCC, we performed this bioinformatics analysis by establishing an independent prognostic ferroptosis-related gene signature using the cancer genome atlas (TCGA) database and validated it in the ArrayExpress database. Then, common functional annotations in both cohorts were screened out with Gene Ontology (GO), Kyoto Encyclopedia of Genes and Genomes (KEGG) and immune infiltration enrichment analyses to explore the underlying mechanisms.

## Methods

**Fig. 1:**
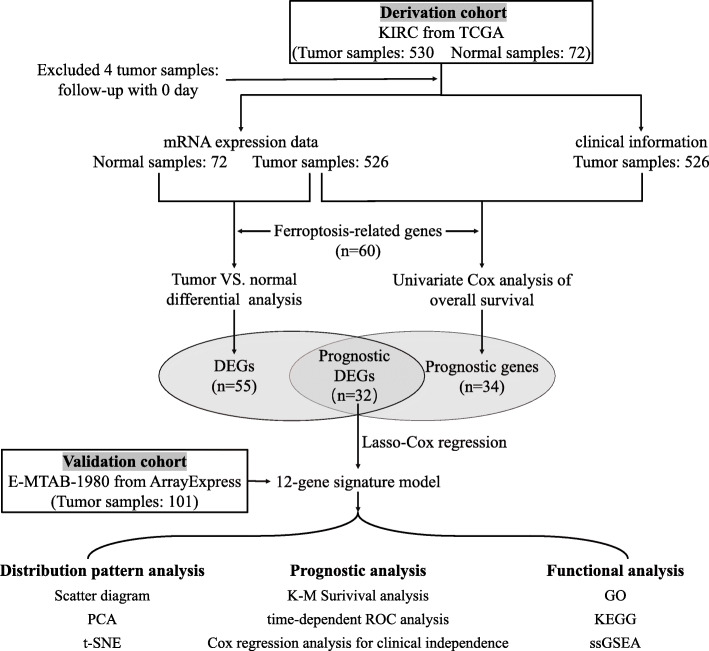
Flow chart of bioinformatics analysis. DEGs, differentially expressed genes; PCA, principal component analysis; t-SNE, t-distributed stochastic neighbour embedding; K-M, Kaplan-Meier; ROC, receiver operating characteristic; GO, Gene Ontology; KEGG, Kyoto Encyclopedia of Genes and Genomes; ssGSEA, single-sample gene set enrichment analysis

The flow chart of the bioinformatics analysis is presented in Fig. 1. All statistical analyses were completed in R language software (Version 4.0.3) [26], and *P* < 0.05 was considered statistically significant without a specified setting.

### Acquisition of ferroptosis-related genes and ccRCC cohorts

A comprehensive literature survey about ferroptosis was performed, and high-quality articles were retrieved, in which 60 ferroptosis-related genes were identified and are presented in Supplementary Table S1 [27–30].

The derivation set and validation set of ccRCC were retrieved from the KIRC cohort (including 526 ccRCC tissue samples and 72 normal kidney tissue samples) in the TCGA database (https://portal.gdc.cancer.gov/repository) and accession E-MTAB-1980 (including 101 ccRCC tissue samples) in the ArrayExpress database (www.https://www.ebi.ac.uk/arrayexpress), respectively. Both raw count values of gene expression and clinical information were downloaded from the corresponding databases. The gene expression profiles of the KIRC and E-MTAB-1980 cohorts were normalized with the “edgeR” package [31] in R language software. Patients with 0 follow-up days were removed from further analysis. Since all retrieved data were from public databases, no ethical review or approval from an Ethics Committee was required. We identified differentially expressed ferroptosis-related genes with prognostic value between ccRCC tissues and normal kidney tissues.

### Identifying differentially expressed ferroptosis-related genes with prognostic value between ccRCC tissues and normal kidney tissues

The gene expression profiles of the KIRC cohort were subjected to differential expression analysis using the “edgeR” R package. Differentially expressed genes (DEGs) between ccRCC tissues and normal kidney tissues were screened out with a false discovery rate (FDR) < 0.05 and |fold change (FC)| > 1. DEGs related to ferroptosis were selected and demonstrated with a heat map generated by the “pheatmap” R package [32]. Then, univariate Cox analysis of overall survival (OS) for 60 ferroptosis-related genes was performed to identify the genes with prognostic value. A Cox *P*-value < 0.05 indicated a significant relationship to OS. Ferroptosis-related DEGs with prognostic values were selected using intersection analysis of DEGs and prognostically valuable genes with the “Venn” R package [33] and then visualized with a protein-protein interaction (PPI) network generated by the STRING database (version 11.0) [34] and a correlation network generated by the “igraph” [35] and the “reshape2” [36] R packages.

### Construction and validation of a prognostic ferroptosis-related gene signature

To minimize the risk of overfitting, we used Lasso-penalized Cox regression analysis to rule out genes with an overfitting tendency and construct a prognostic signature with the “glmnet” R package [37–39]. The risk scores in the derivation set and the validation set were calculated according to a linear combination of the normalized expression value of each prognostic ferroptosis-related DEG and its corresponding multivariate Cox regression coefficient (β). The risk score calculation formula was as follows: Risk score = β × expression value of CARS1 + β × expression value of HMGCR + β × expression value of CHAC1 + β × expression value of GOT1 + β × expression value of CD44 + β × expression value of STEAP3 + β × expression value of AKR1C1 + β × expression value of CBS + β × expression value of DPP4 + β × expression value of FANCD2 + β × expression value of SLC1A5 + β × expression value of NCOA4. In both sets, each patient was given a risk score from the calculation of the formula and then assigned to either high- or low-risk group divided by the median risk score of the derivation set. The distribution patterns were described for the risk scores and the corresponding survival times of all patients with scatter diagrams by the “pheatmap” R package, the gene expression of established signature with principal component analysis (PCA) by the “stats” R package [26], and the patients in different risk groups with t-distributed stochastic neighbour embedding (t-SNE) by the “Rtsne” R package [40]. Kaplan-Meier (K-M) survival analysis and time-dependent receiver operating characteristic (ROC) analysis based on OS were performed using the “survival” package [41], the “survminer” package [42] and the “timeROC” package [43] in R to estimate the prognostic accuracy of the gene signature in the derivation set and verify it in the validation set.

### Prognostic independence of the gene signature from traditional clinical characteristics

To further assess the independent prognostic value of the established gene signature, we used univariate and multivariate Cox regression analyses to determine whether it was affected by other clinical characteristics. Several available clinical characteristics, including age, gender and TNM stage, were transformed into dichotomous variables and included for the calculation of hazard ratios (HRs) and 95% confidence intervals (CIs) based on OS. *P* < 0.05 was considered statistically significant.

### GO, KEGG and immune infiltration enrichment analyses for risk-related DEGs

According to the risk grouping, normalized gene expression matrixes of the derivation set and the validation set generated above were applied with the “limma” R package [44] to identify risk-related DEGs with the cut-off criteria of |FC| ≥ 1.5 and FDR < 0.05, respectively. Risk-related DEGs were analysed with GO [45] and KEGG [46] using the “clusterProfiler” R package [47]. The top 30 enriched terms in 3 categories of GO (including biological process (BP), cellular component (CC) and molecular function (MF)) and KEGG with the cut-off criteria of gene count > 10 and *P*-value < 0.05 in both 2 sets were intersected to obtain the overlapping enriched terms. Then, single-sample gene set enrichment analysis (ssGSEA) [48] for immune infiltration was applied with the “GSVA” R package [49] to assess the infiltration score of 16 immune cells and the activity of 13 immune-related functions. With the annotated gene sets provided in Supplementary Table S2, we quantified the immune infiltration enrichment scores for different immune cells and immune-related functions to further investigate the correlation between the risk score and immune status.

## Results

### Identification of prognostic ferroptosis-related DEGs in the KIRC cohort

**Fig. 2:**
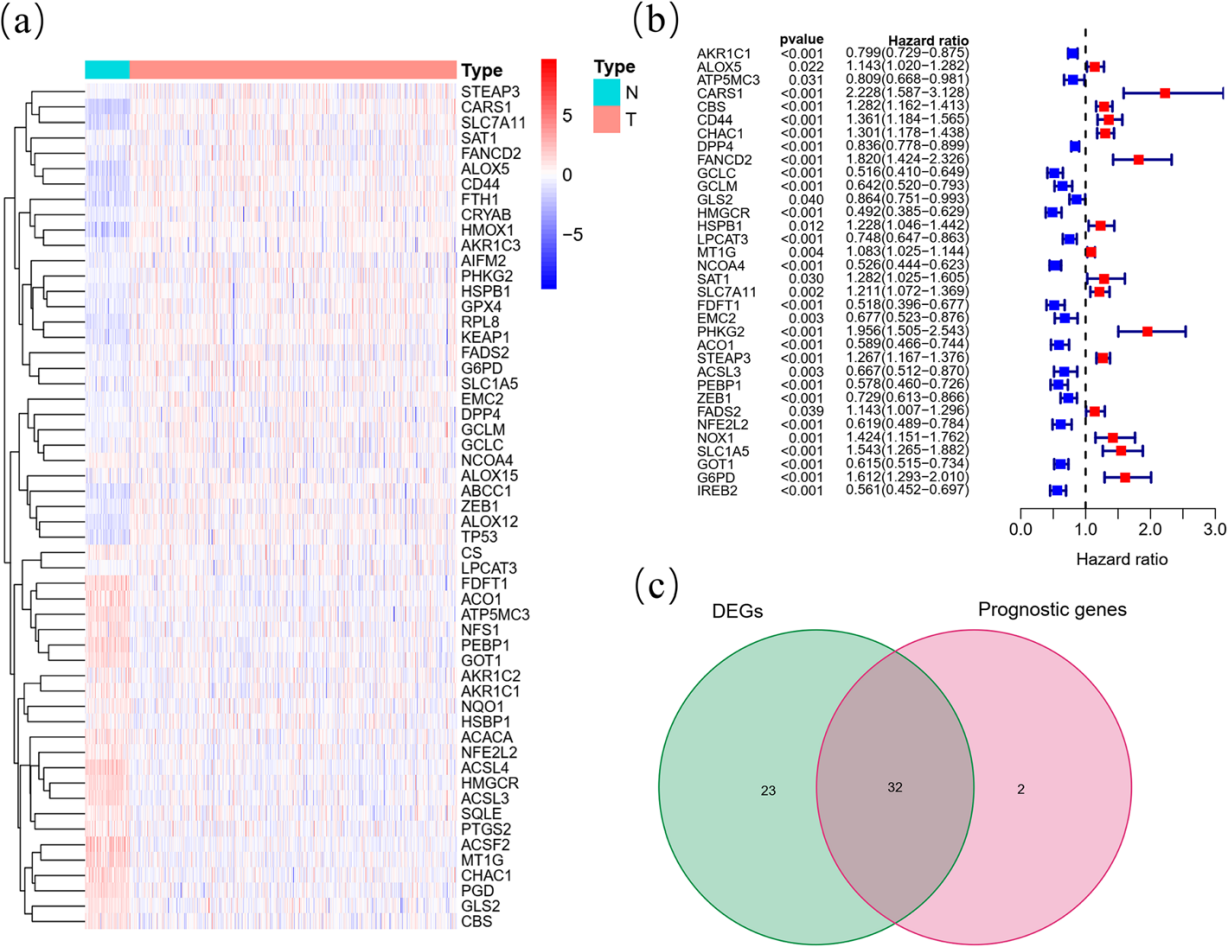
Identification of the prognostic ferroptosis-related DEGs in the KIRC cohort. (**A**) Heatmap showing the ferroptosis-related DEGs identified with differential expression analysis. (**B**) Forest plots showing the significantly prognostic ferroptosis-related genes identified with univariate Cox regression analysis based on OS. (**C**) Venn diagram showing the overlapping genes between ferroptosis-related DEGs and OS-correlated genes

**Fig. 3:**
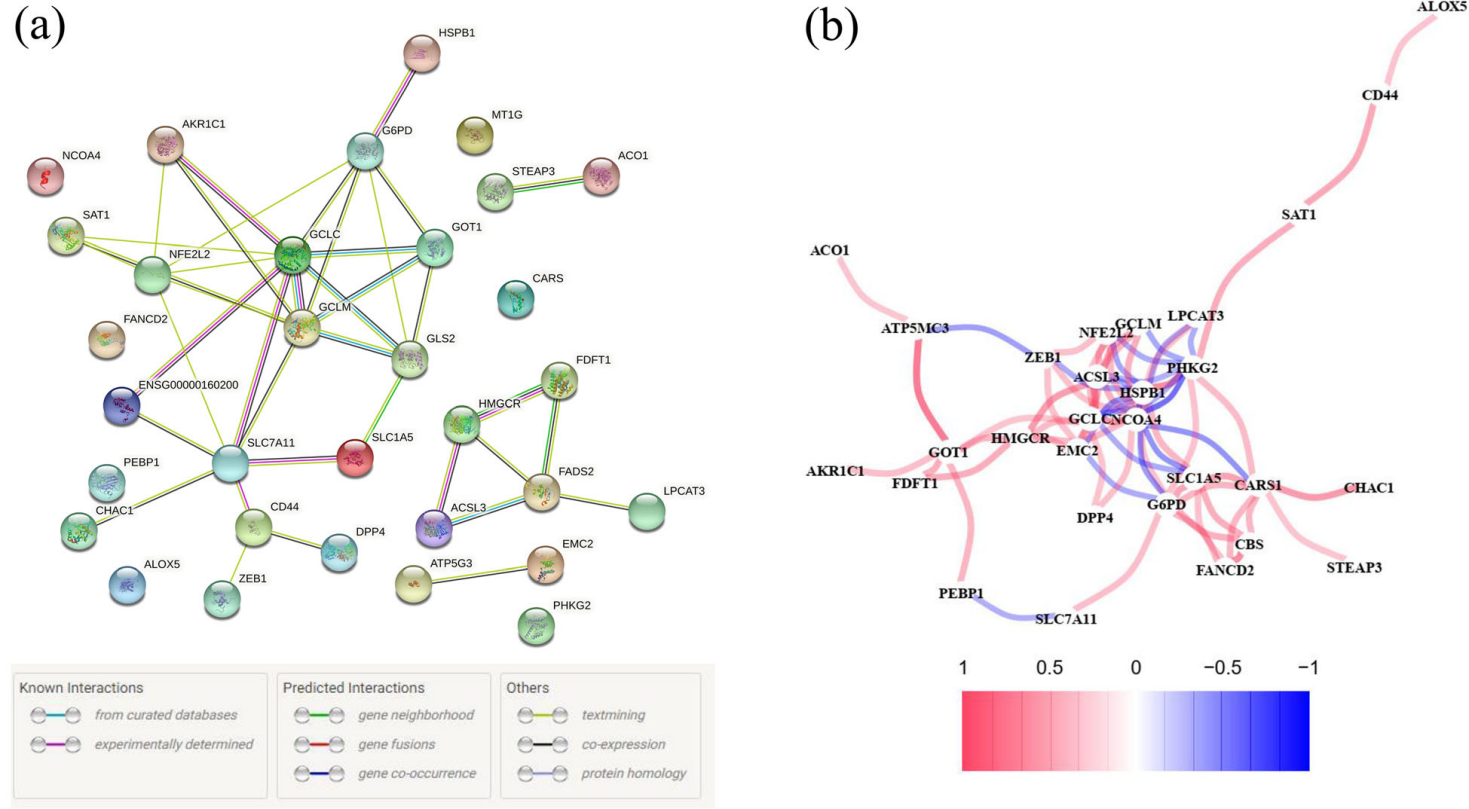
Interaction networks of the prognostic ferroptosis-related DEGs in the KIRC cohort. (**A**) The PPI network downloaded from the STRING database indicated the interactions among candidate genes. (**B**) The correlation network of candidate genes, in which the correlation coefficients are represented by different colours

Among 60 ferroptosis-related genes, 55 (91.67%) were differentially expressed between ccRCC samples and normal kidney samples (Fig. 2A), and 34 (56.67%) were considered OS-related in the univariate Cox regression analysis (Fig. 2B), from which 32 overlapping genes correlated to ferroptosis and OS were selected by the intersection analysis (Fig. 2C). Interactions of 32 prognostic ferroptosis-related DEGs were further visualized with the PPI and correlation networks (Fig. 3A-B).

### Establishment of a ferroptosis-related 12-gene signature in the KIRC cohort

**Fig. 4:**
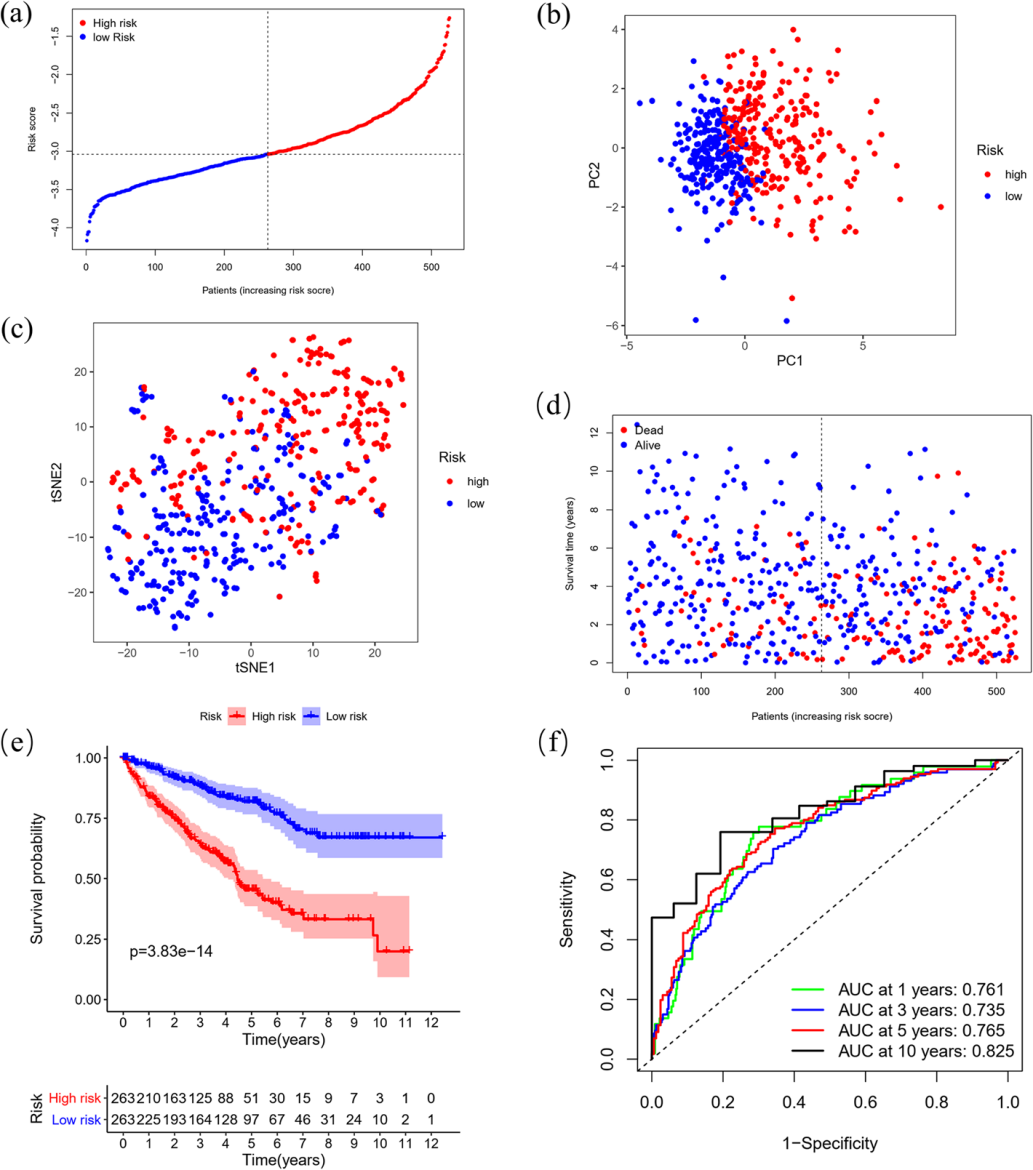
Distribution and prognostic analyses of the 12-gene signature in the KIRC cohort. (**A**) The distribution and median value of the risk scores in the KIRC cohort. (**B**) PCA plot of the KIRC cohort showing the distribution of the established gene signature expression in different risk groups. (**C**) t-SNE plot of the KIRC cohort showing the distribution of the patients in different risk groups. (**D**) The distributions of the risk scores and corresponding survival times of all patients in the KIRC cohort. (**E**) OS-based K-M survival curves for the patients in the high-risk group and low-risk group in the KIRC cohort. (**F**) AUC of time-dependent ROC curves verified the prognostic performance of the risk score in the KIRC cohort

The 32 prognostic ferroptosis-related DEGs were subjected to Lasso Cox regression analysis based on OS, and a 12-gene signature with CARS1, HMGCR, CHAC1, GOT1, CD44, STEAP3, AKR1C1, CBS, DPP4, FANCD2, SLC1A5 and NCOA4 was identified in the KIRC cohort. According to the median risk score, patients were divided into a high-risk group (*n* = 263) and a low-risk group (*n* = 263) (Fig. 4A), which were distributed into two sets in PCA and t-SNE (Fig. 4B-C). In addition, considering survival outcomes, we observed that the high-risk group had more deaths than the low-risk group (Fig. 4D). To further evaluate the prognostic value and predictive performance of the gene signature, we performed K-M survival and time-dependent ROC analyses, and both produced significant results. The K-M survival curve showed significantly worse survival outcomes for patients in the high-risk group than for patients in the low-risk group (*P* = 3.83e-14) (Fig. 4E), and the area under the curve (AUC) reached 0.761 at 1 year, 0.735 at 3 years, 0.765 at 5 years, and 0.825 at 10 years in the ROC analysis (Fig. 4F).

### Validation of the prognostic predictive performance for the 12-gene signature in the E-MTAB-1980 cohort

**Fig. 5:**
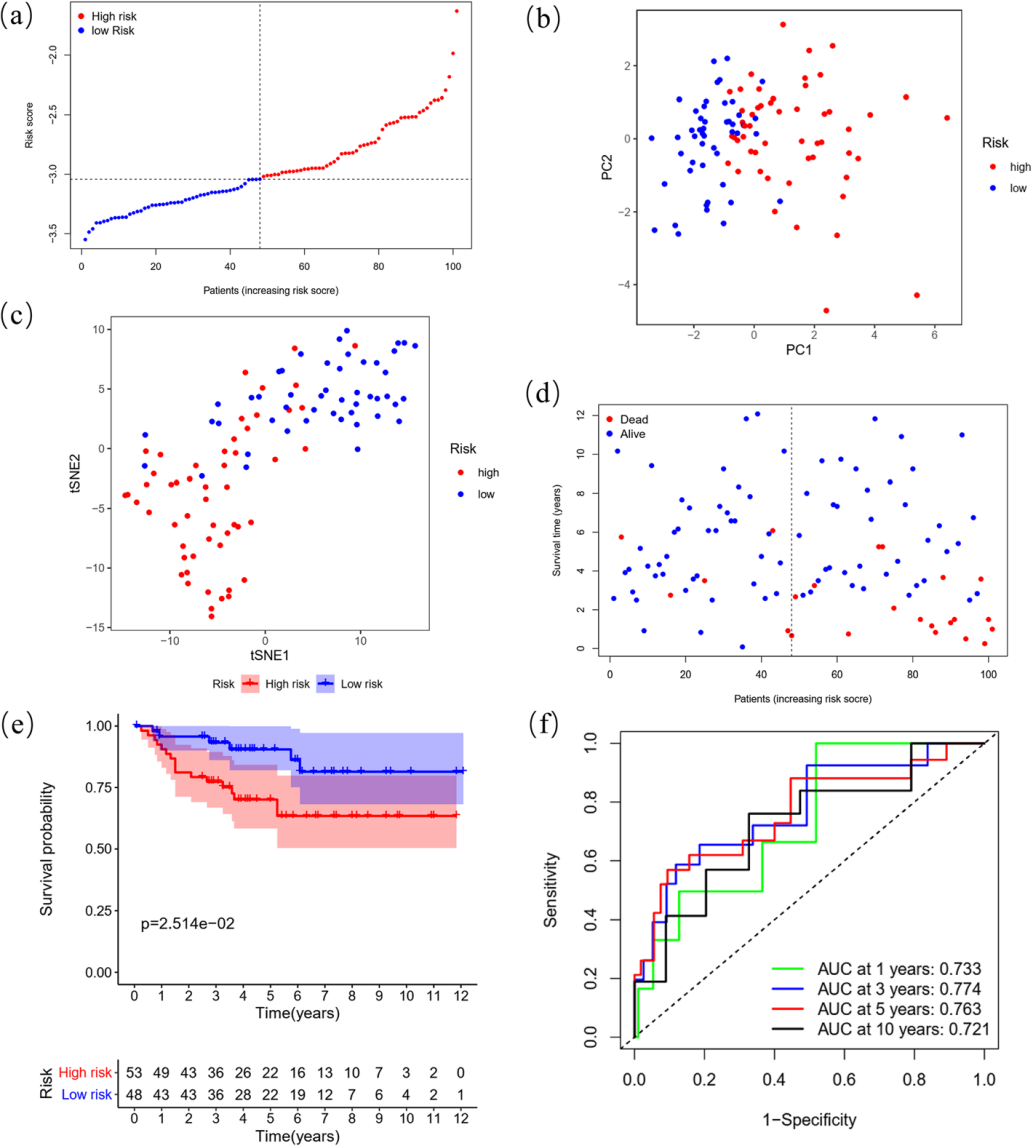
Distribution and prognostic analyses of the 12-gene signature in the E-MTAB-1980 cohort. (**A**) The distribution and median value of the risk scores in the E-MTAB-1980 cohort. (**B**) PCA plot of the E-MTAB-1980 cohort showing the distribution of the established gene signature expression in different risk groups. (**C**) t-SNE plot of the E-MTAB-1980 cohort showing the distribution of the patients in different risk groups. (**D**) The distributions of the risk scores and corresponding survival times of all patients in the E-MTAB-1980 cohort. (**E**) OS-based K-M survival curves for the patients in the high-risk group and low-risk group in the E-MTAB-1980 cohort. (**F**) AUC of time-dependent ROC curves verified the prognostic performance of the risk score in the E-MTAB-1980 cohort

By applying the KIRC median risk score in the E-MTAB-1980 cohort, we categorized 101 patients as either high-risk (*n* = 53) or low-risk (*n* = 48) with different PCA, t-SNE and death probability distributions similar to the KIRC cohort (Fig. 5A-D). Consistently, a positive risk-related K-M survival curve with a significant *P*-value (2.514e-2) and ROC curves with considerable AUCs (0.733 at 1 year, 0.774 at 3 years, 0.763 at 5 years, and 0.721 at 10 years) were also established as convincing validation for the gene signature (Fig. 5E-F).

### Prognostic independence of the 12-gene signature from clinical characteristics

**Fig. 6:**
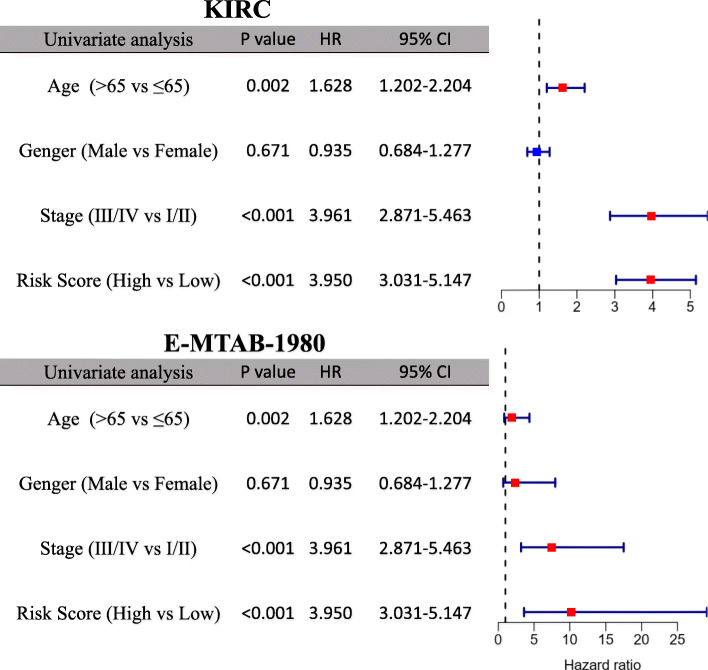
Univariate Cox regression analysis regarding OS in the KIRC and E-MTAB-1980 cohorts

**Fig. 7:**
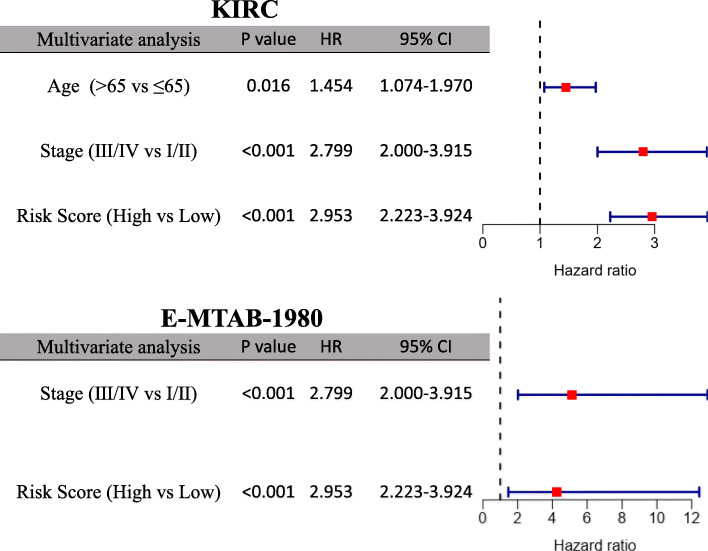
Multivariate Cox regression analysis regarding OS in the KIRC and E-MTAB-1980 cohorts

Age, gender and TNM stage were included to test the independence of the prognostic 12-gene signature in the univariate and multivariate Cox regression. As shown in the univariate Cox regression analysis, risk score and TNM stage were proven to be strong OS-related factors in both the KIRC (risk score: HR = 3.950, 95% CI = 3.031–5.147, *P* < 0.001; TNM stage: HR = 3.961, 95% CI = 2.871–5.463, *P* < 0.001) and the E-MTAB-1980 cohorts (risk score: HR = 10.247, 95% CI = 3.604–29.136, *P* < 0.001; TNM stage: HR = 7.472, 95% CI = 3.191–17.496, *P* < 0.001), as well as age in the KIRC cohort (HR = 1.628, 95% CI = 1.202–2.204, *P* = 0.002) (Fig. 6). After independent correction for other clinical characteristics in the multivariate Cox regression, the risk score was still a solid prognostic predictor for OS in both cohorts (KIRC: HR = 2.953, 95% CI = 2.223–3.924, *P* < 0.001; E-MTAB-1980: HR = 4.270, 95% CI = 1.465–12.439, *P* = 0.008) (Fig. 7).

### GO and KEGG enrichment analyses in the KIRC and E-MTAB-1980 cohorts

**Fig. 8:**
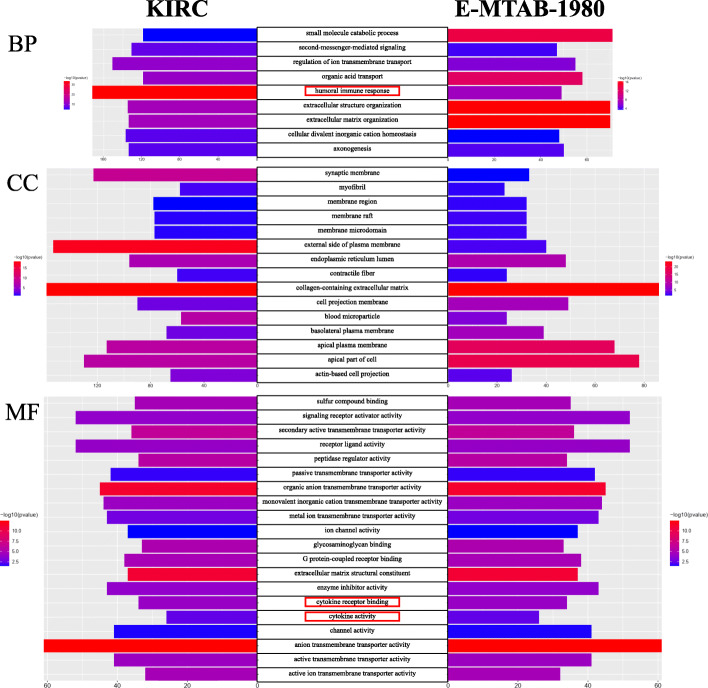
Results of GO enrichment analysis. The 9, 15 and 20 overlapping terms of the top 30 en-riched BP, CC and MF terms between the KIRC and E-MTAB-1980 cohorts are displayed, respectively. The red rectangles indicate the immune-related terms

**Fig. 9:**
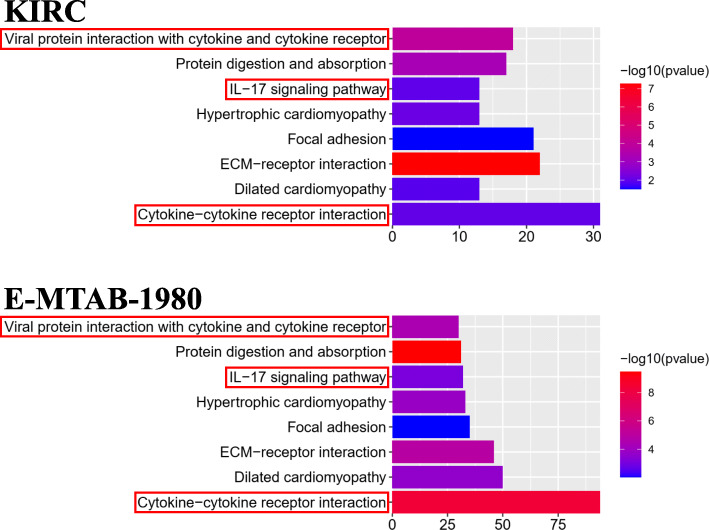
Results of KEGG enrichment analysis. The 8 overlapping terms of the top 30 enriched KEGG terms between the KIRC and E-MTAB-1980 cohorts are displayed. The red rectangles indicate the immune-related terms

After differential expression analysis between the high- and low-risk groups, 8597 DEGs significantly enriched in 958 BP, 92 CC, 155 MF and 66 KEGG terms were identified in the KIRC cohort. For the E-MTAB-1980 cohort, 1253 DEGs were significantly enriched in 605 BP, 50 CC, 76 MF and 28 KEGG terms. All significantly enriched terms are shown in Supplementary Tables S3 and S4 for the KIRC and E-MTAB-1980 cohorts, respectively. We selected the top 30 enriched terms in GO and KEGG in both cohorts and found 9, 15, 20 and 8 overlapping enriched terms in BP, CC, MF and KEGG, respectively (Figs. 8-9). As expected, several iron-related molecular functions, including metal ion transmembrane transporter activity, ion channel activity, and active ion transmembrane transporter activity, were identified. Moreover, several immune-related terms in BP (humoral immune response), MF (cytokine receptor binding; cytokine activity) and KEGG (viral protein interaction with cytokine and cytokine receptor; IL-17 signaling pathway; cytokine-cytokine receptor interaction) were significantly enriched in both cohorts (Figs. 8-9).

### Immune infiltration ssGSEA in the KIRC and E-MTAB-1980 cohorts

**Fig. 10:**
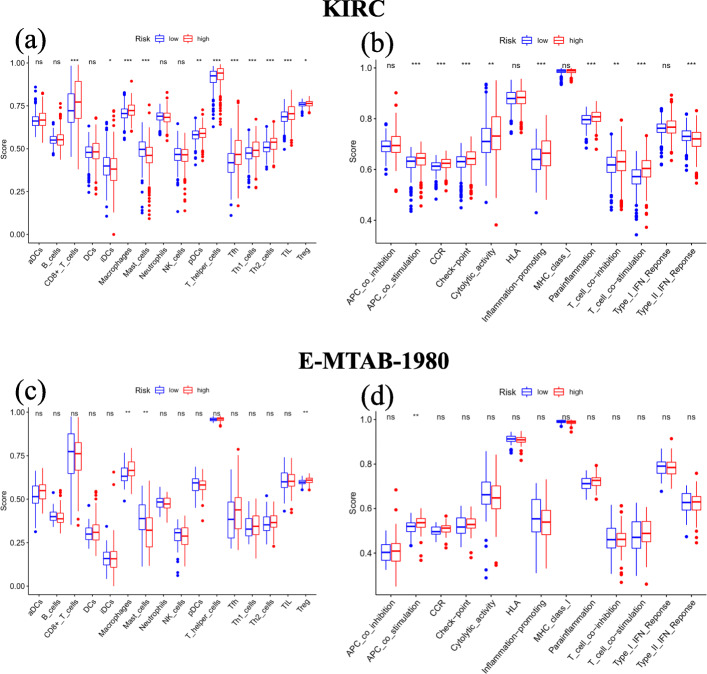
Results of ssGSEA immune infiltration in the KIRC and E-MTAB-1980 cohorts. (**A**) The ssGSEA scores of 16 immune cells between different risk groups in the KIRC cohort. (**B**) The ssGSEA scores of 13 immune-related functions between different risk groups in the KIRC cohort. (**C**) The ssGSEA scores of 16 immune cells between different risk groups in the E-MTAB-1980 co-hort. (**D**) The ssGSEA scores of 13 immune-related functions between different risk groups in the E-MTAB-1980 cohort. aDCs, activated dendritic cells; DCs, dendritic cells; iDCs, immature den-dritic cells; NK cells, natural killer cells; pDCs, plasmacytoid dendritic cells; Tfh, follicular helper T cell; Th1 cells, helper T cells 1; Th2 cells, helper T cells 2; TIL, tumour infiltrating lymphocyte; Treg, regulatory T cell; CCR, cytokine-cytokine receptor; HLA, human leukocyte antigen; MHC, major histocompatibility complex; IFN, immune interferon; ns, not significant; *, adjusted *P* < 0.05; **, adjusted *P* < 0.01; ***, adjusted *P* < 0.001

In the KIRC cohort, we found that 9 out of 16 immune cells had significantly higher infiltration enrichment scores in the high-risk group: CD8+ T cells, macrophages, plasmacytoid dendritic cells (pDCs), T helper cells, follicular helper T cell (Tfh), helper T cells 1 (Th1 cells), helper T cells 2 (Th2 cells), tumour infiltrating lymphocyte (TIL) and regulatory T cell (Treg), while immature dendritic cells (iDCs) and mast cells showed the opposite pattern (Fig. 10A). Similarly, the high-risk group was predicted to be significantly correlated with most immune-related functions, except for the Type II immune interferon (IFN) Response (Fig. 10B). Regarding ssGSEA in the E-MTAB-1980 cohort, we validated the significantly different infiltration scores of 3 immune cells (macrophages, mast cells and Tregs) and 1 immune-related function (antigen processing cell (APC) co-stimulation) (Fig. 10C-D).

## Discussion

In the current study, the expression and clinical data were retrieved from the KIRC cohort in TCGA and the E-MTAB-1980 cohort in ArrayExpress. Within 60 ferroptosis-related genes, we performed differential expression analysis and univariate Cox analysis to screen 32 prognostic DEGs, from which Lasso-penalized Cox regression analysis was applied to construct a prognostic 12-gene signature with CARS1, HMGCR, CHAC1, GOT1, CD44, STEAP3, AKR1C1, CBS, DPP4, FANCD2, SLC1A5 and NCOA4. The signature-correlated risk score of each patient in both cohorts was calculated, according to which patients were assigned to either the high- or low-risk group divided by the median risk score of the KIRC cohort. Then, the independent prognostic value of the signature was further assessed by K-M survival, ROC and Cox regression analyses in the KIRC cohort and validated in the E-MTAB-1980 cohort. Finally, risk-related DEGs were identified in both cohorts and subjected to enrichment analyses for GO, KEGG and immune infiltration. As expected, several iron-related GO and KEGG terms were significantly enriched. However, interestingly, some immune-related terms were identified. Further immune infiltration analysis showed that 3 immune cells and 1 immune-related function were enriched in both cohorts, which supported the potential relationship between tumour immunity and ferroptosis in ccRCC.

The prognostic 12-ferroptosis-related-gene signature contains 5 protective genes (HMGCR, GOT1, AKR1C1, DPP4 and NCOA4) and 7 risk genes (CARS1, CHAC1, CD44, STEAP3, CBS, FANCD2 and SLC1A5), which can be classified as iron metabolism-related (NCOA4, STEAP3 and FANCD2), lipid metabolism-related (HMGCR, AKR1C1 and DPP4), (anti) oxidant metabolism-related (CHAC1, CD44, CBS and CARS1) and energy metabolism-related genes (GOT1 and SLC1A5) according to the potential gene-regulating function for ferroptosis [30].

In iron metabolism, NCOA4 can help elevate the levels of free iron by recruiting iron-storage protein ferritin (FTH) including ferritin light chain (FTL) and ferritin heavy chain 1 (FTH1) for lysosomal degradation and then releasing iron. As a participant in free radical formation and lipid peroxidation propagation, the accumulation of iron can increase the ferroptotic sensitivity of cells. Therefore, inhibition of NCOA4 can suppress ferroptosis induced by amino acid/cystine deprivation or erastin [50, 51]. In addition, the expression STEAP3, a metalloreductase reducing Fe3+ to Fe2+, can also be upregulated in ferroptosis. In the endosome, Fe2+ reduced by STEAP3 will be released into the cytosol to increase free iron and therefore participate in ferroptosis. In contrast to what was mentioned above, FANCD2 is a nuclear protein involved in DNA damage repair with a potential ability to decrease iron levels. In bone marrow stromal cells, the knockout of FANCD2 increased the expression of STEAP3 and enhanced erastin-induced ferroptosis [52].

HMGCR is a reductase that can catalyse 3-hydroxy-3-methyl-glutaryl coenzyme A (HMG-CoA) to synthesize mevalonic acid and then participate in the synthesis of sterol coenzyme Q10 (CoQ10), an endogenous suppressor of ferroptosis. A study showed that the drug inhibition of HMGCR is responsible for the enhancement of FIN56-induced ferroptosis [53]. AKR1C1 is a member of aldosterone reductase family 1 (AKR1), an aldehyde detoxification enzyme family that is involved in steroid metabolism. The overexpression of AKR1C (including AKR1C1, AKR1C2 and AKR1C3) has been proven to have an antiferroptotic effect through the reduction reaction converting the end products of lipid peroxides to the corresponding nontoxic lipid-derived alcohols [54]. DPP4 is a binding protein to NOX, a participant in a membrane-bound enzyme complex that produces downstream ROS. The combination of NOX-DPP4/CD26 can cause plasma membrane lipid peroxidation and therefore result in ferroptosis, which can be blocked by p53 through DPP4 silencing in colorectal cancer cells [55]. In addition, the involvement of DPP4 and p53 was observed in Golgi stress-induced ferroptosis [56].

During (anti) oxidant metabolism in ferroptosis, cysteine serves as an initiator by providing materials for the biosynthesis of glutathione (GSH), which contributes an antiferroptotic effect. Extracellular cysteine can be transported into the cytosol by exchange with intracellular glutamate through the cysteine-glutamate exchange system Xc-. CHAC1 and CD44 have been suggested to interact with system Xc- and provide a proferroptotic effect in Burkitt’s lymphoma [57] and an antiferroptosis effect in human gastrointestinal cancer [17], respectively. In another way, homocysteine has an alternative transsulfuration pathway that produces cystathionine promoted by CBS and then cysteine promoted by cystathionine (CTH). In the cytoplasm, cysteine can be charged with tRNACys, which are catalysed by CARS1 and therefore result in a decrease in cysteine. A study showed that the knockdown of CARS1 can increase the compensatory transsulfuration pathway to increase cysteine and suppress ferroptosis induced by erastin, which can be resensitized by silencing CBS [58]. Additionally, the ferroptosis-enhancing effect of suppressing CBS has been demonstrated in hepatocellular carcinoma cells [59].

GOT1 and SLC1A5 are both involved in the energy-metabolic network for ROS production in ferroptosis. In cystine deprivation- or erastin-induced ferroptosis, SLC1A5-mediated L-glutamine uptake is a critical process for the production of glutamate, which is further converted into α-ketoglutarate (αKG) by transaminase GOT1-mediated transamination [60]. The accumulation of αKG can be converted into acetyl coenzyme A (acetyl-CoA) in the cytoplasm for lipid biosynthesis and fatty acid synthesis or increase mitochondrial ROS and iron levels to promote ferroptosis [61, 62]. Immune cells are attracted and accumulated by a set of signals to help program cell death during apoptosis [63]. It is conceivable that similar signal patterns will attract APCs and other immune cells to assist the accomplishment of ferroptosis, although solid proof is still lacking. However, an in vitro study on macrophage clearance of ferroptotic cells supported this possibility [64]. Bioinformatically, several studies have demonstrated the potential connection between RCC and immune infiltration [65, 66]. Clinically, in addition to palliative targeted therapy, considerable promising results of monotherapy with novel immunotherapies, such as immune checkpoint inhibitors (ICIs), have been observed in some advanced RCC patients [67]. Moreover, a combinatory ICI therapy of nivolumab plus ipilimumab has been approved for the phase-3 clinical trial last year [68].

In the present study, with immune annotation analysis based on risk groups, we discovered that macrophages, mast cells, Tregs and immune-related function APC co-stimulation were significantly enriched in both cohorts, which indicates a potential underlying modulation between tumour immunity and ferroptosis in ccRCC. Macrophages, mast cells and Tregs are all APCs that are capable of presenting processed antigens to T cells and activating the immune response by co-stimulation. Tumour-associated macrophages (TAMs) have a dual character and have either procancer or anticancer effects in the immune system [69, 70]. As in ccRCC, it has been demonstrated that an increased density of TAMs is associated with poor clinical prognosis and aggressive tumour migration [71, 72]. Similarly, Tregs show tumour-facilitating potential in ccRCC. Tregs have been proven to have an association with worse prognosis in ccRCC [73, 74]. For mast cells, the research of Şenbabaoğlu et al. revealed that mast cell density has an independent negative correlation with OS and progression-free survival (PFS) in ccRCC [66]. Moreover, Fu et al. observed that mast cells were independently negatively correlated with cancer-specific survival (CSS) and relapse-free survival (RFS) in ccRCC [75]. In addition, in vitro and in vivo experiments have demonstrated the angiogenesis-promoting effect of mast cells in RCC [76]. Although multiple pieces of evidence have elucidated the functions of macrophages, mast cells and Tregs in ccRCC, the underlying mechanism remains poorly characterized, and this issue in the field of ferroptosis is lacking. The relationships between the immune response and ferroptosis and how they correlate with prognosis in ccRCC still require further investigation.

Several limitations were observed in the present study. As a bioinformatics analysis, the weakness of lacking experimental and clinical validation is inevitable, as well as the various possible results from using different cut-off criteria, statistical methods or analysis tools. Additionally, establishing a prognostic model by considering a single hallmark might lead to the regrettable absence of many other promising prognostic genes.

In summary, we established a novel ferroptosis-related 12-gene signature that was proven to be an independent prognostic predictor for OS in ccRCC. Through functional annotation analyses, the gene signature was shown to be tumour immunity-correlated; however, the underlying mechanism is still poorly characterized and needs further exploration.

## Supplementary Information

**Additional file 1.**

## Data Availability

The datasets analyzed for this study can be found in the KIRC project in TCGA database (https://portal.gdc.cancer.gov/repository) and E-MTAB-1980 cohort in ArrayExpress database (https://www.ebi.ac.uk/arrayexpress/experiments/E-MTAB-1980/). The literature survey data from this study can be request from the corresponding author Peilin Shen.

## Abbreviations

acetyl-CoA: acetyl coenzyme A
αKG: α-ketoglutarate
AKR1: aldosterone reductase family 1
APC: antigen processing cell
AUC: area under the curve
BP: biological process
CC: cellular component
ccRCC: clear cell renal cell carcinoma
CIs: confidence intervals
CoQ10: coenzyme Q10
CSS: cancer-specific survival
CTH: cystathionine
DEGs: differentially expressed genes
FC: fold change
FDR: false discovery rate
FTH: ferritin
FTH1: ferritin heavy chain 1
FTL: ferritin light chain
GO: Gene Ontology
GSH: glutathione
HMG-CoA: 3-hydroxy-3-methyl-glutaryl coenzyme A
HRs: Hazard ratios
ICIs: immune checkpoint inhibitors
iDCs: immature dendritic cells
IFN: immune interferon
KEGG: Kyoto Encyclopedia of Genes and Genomes
K-M: Kaplan-Meier
MF: molecular function
mTOR: mammalian target of rapamycin
OS: overall survival
PCA: principal component analysis
pDCs: plasmacytoid dendritic cells
PFS: progression-free survival
PPI: protein-protein interaction
RCC: renal cell carcinoma
RCD: regulated cell death
RFS: relapse-free survival
ROC: receiver operating characteristic
ROS: reactive oxygen species
ssGSEA: single-sample gene set enrichment analysis
TAMs: Tumour-associated macrophages
TCGA: the cancer genome atlas
Tfh: follicular helper T cell
Th1 cells: helper T cells 1
Th2 cells: helper T cells 2
TIL: tumour infiltrating lymphocyte
TKIs: tyrosine kinase inhibitors
Treg: regulatory T cell
t-SNE: t-distributed stochastic neighbour embedding
